# Effects of Post-Curing Light Intensity on the Mechanical Properties and Three-Dimensional Printing Accuracy of Interim Dental Material

**DOI:** 10.3390/ma15196889

**Published:** 2022-10-04

**Authors:** Min-Jung Kang, Jung-Hwa Lim, Chan-Gyu Lee, Jong-Eun Kim

**Affiliations:** Department of Prosthodontics, Yonsei University College of Dentistry, 50-1 Yonsei-ro, Seodaemun-gu, Seoul 03722, Korea

**Keywords:** additive manufacturing, three-dimensional printing, post-curing, CAD/CAM, flexural strength, Vickers microhardness, three-dimensional accuracy, degree of conversion

## Abstract

This study evaluated the effects of the light intensity of curing and the post-curing duration on the mechanical properties and accuracy of the interim dental material. After designing the specimen, 3D printing was performed, and the light intensity was divided into groups G20, G60, G80, and G120 (corresponding to 1.4–1.6, 2.2–3.0, 3.8–4.4, and 6.4–7.0 mW/cm^2^, respectively), with no post-curing or 5, 10, or 20 min of post-curing being performed. The flexural properties, Vickers microhardness, degree of conversion (DC), and 3D accuracy were then evaluated. The flexural properties and Vickers microhardness showed a sharp increase at the beginning of the post-curing and then tended to increase gradually as the light intensity and post-curing time increased (*p* < 0.001). On the other hand, there was no significant difference between groups in the accuracy analysis of a 3D-printed three-unit bridge. These results indicate that the light intensity of the post-curing equipment influences the final mechanical properties of 3D-printed resin and that post-curing can be made more efficient by optimizing the light intensity and post-curing time.

## 1. Introduction

With the recent development of computer-aided design/computer-aided manufacturing (CAD/CAM) systems for dentistry, digital data can be acquired using oral or model scanners, and prostheses can be analyzed and designed using CAD software. Methods of fabricating the designed prostheses are broadly divided into additive manufacturing (AM) and subtractive manufacturing (SM). The AM represented by three-dimensional (3D) printing has various advantages compared to the SM represented by milling. There is no unnecessary consumption of cut and discarded material, and it is not affected by the shape or size of the cutting tool, making it easy to reproduce complex shapes and perform mass production [1,2]. Three-dimensional printing technology has been increasingly used in dental clinics and is now widely used for various purposes, such as manufacturing diagnostic casts, surgical guides, temporary restorations, occlusal splints, and complete dentures [3,4,5].

The 3D printing method commonly used in dentistry includes the digital light processing (DLP) and stereo lithography apparatus (SLA) methods of photocuring liquid-type resins. If the SLA method completes the layer by irradiating the ultraviolet (UV) laser beam on the tank that contains the polymer and curing only the necessary parts, the DLP method cures the entire layer at once with a UV lamp and dimming device as if using the camera flash. Both the photocuring process using UV light (100–400 nm) is performed, and when free radicals are formed using a photo-initiator, a radical-reactive monomer or oligomer is cured through a continuous reaction. However, since unreacted monomers that are not completely polymerized are present immediately after the 3D printing, washing and post-curing processes must be performed to improve mechanical properties and biocompatibility of outcomes [6].

Unlike diagnostic casts and castable patterns used outside the oral cavity, interim dental materials must function for a certain period and have sufficient strength to withstand masticatory forces, reproduce accurate settling and occlusion in the oral cavity, and be biocompatible. Previous studies have revealed that various parameters during the 3D printing process, such as build angle, support configuration, and layer thickness, can affect the mechanical properties and dimensional accuracy of the resulting product [7,8]. Research has recently been conducted on how post-curing conditions affect flexural properties, surface hardness, accuracy, color tone, and biocompatibility. As the post-curing time and temperature increase, the degree of conversion (DC) has been found to improve, and mechanical properties such as flexural strength (FS) and Vickers microhardness were also improved [9].

Post-curing equipment and dental materials from various manufacturers are currently being used, and differences in DC, surface hardness, and cell viability of 3D printing resin have been found to vary depending on the type and main wavelength of the post-curing equipment [10,11]. Previous studies indicated that using a higher curing-light intensity for a conventional light-curing or dual-curing composite resin made the polymerization more immediate and deeper, but greater shrinkage decreased the marginal adaptation of the prostheses [12]. However, there have been few research results related to DLP 3D printers and 3D printing resin polymerizing in a layer-by-layer manner. As far as the author was aware, there was also a lack of research results on how the mechanical properties, DC, and dimensional accuracy of 3D-printed products vary with the post-curing time and UV light intensity during the post-curing process. Therefore, guidelines for light intensity and post-curing time are needed to obtain optimal physical properties.

The purpose of this study was therefore to determine the effect of UV light intensity on the mechanical properties and accuracy of interim dental materials during the post-curing process. The null hypothesis of this study was that the light intensity of the post-curing equipment and post-curing time do not affect the Vickers microhardness (VHN), flexural properties, DC, or dimensional accuracy of the three-unit bridge.

## 2. Materials and Methods

### 2.1. Preparation of 3D Printed Specimens

**Figure 1:**
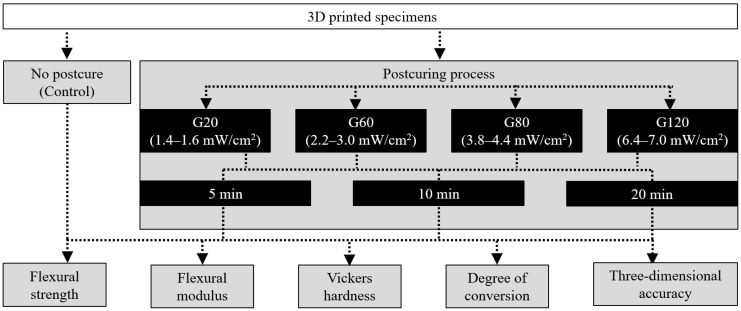
Workflow of the overall experimental process, showing the post-curing conditions and experiment types. FS, flexural strength; FM, flexural modulus; VHN, Vickers microhardness; DC, degree of conversion; 3D, three-dimensional; GS, green state.

The overall experimental process of this study is presented in Figure 1. The specimens used in the study were designed using standard CAD software (Tinkercad, Autodesk, San Rafael, CA, USA). To test the flexural properties, a bar-shaped specimen (25 mm × 2 mm × 2 mm) was designed according to the ISO 10477 standard, and a disc-shaped specimen (15 mm × 2 mm) was designed to evaluate the VHN and DC. In addition, for 3D accuracy evaluation, dental CAD software (DentalCAD, exocad, Darmstadt, Germany) was used to design a three-unit bridge (#15–#17). The designed file was saved in the Standard Tessellation Language (STL) format, and the output value was set (layer thickness = 50 μm, build direction = 0°). The resin (MAZIC D TEMP, VERICOM, Kang-won, Korea) was used as a material for the crown and bridge, and a DLP-type 3D printer (Asiga Max UV, Asiga, Sydney, Australia), which is available with 365 nm or 405 nm LED, was used. After completing the 3D printing, the specimen was washed for 3 min using a stirrer-type washer (Twin Tornado, MEDIFIVE, Incheon, Korea) and an ultrasonic cleaner (UCP-02, Lab Companion, Billerica, MA, USA) with 90% isopropyl alcohol.

### 2.2. Light Characterization of Post-Curing Equipment

In the post-curing equipment used in this experiment (P-cure, Straumann, Basel, Switzerland), the light intensity could be adjusted in 5 units from 20 to 170%, but the company did not provide clear information on the intensity. Therefore, after measuring each intensity in the pilot study, the light intensity showing a clear difference was adopted as a representative (20, 60, 80, 120%) and used in this experiment. According to the purpose of the study, light intensities of 1.4–1.6, 2.2–3.0, 3.8–4.4, and 6.4–7.0 mW/cm^2^ were applied during the post-curing stage (designated as groups G20, G60, G80, and G120, respectively), and post-curing was performed for 5, 10, and 20 min. The post-curing equipment applied light irradiation to both the upper and lower plates that divided the inside of the chamber using a 385 nm UV LED light source (P-cure, Straumann, Switzerland). In addition, the green-state group (without post-curing) was used as a control. The intensity and temperature inside the post-curing equipment chamber were measured in real time for 20 min for each light intensity group prior to the experiment using a thermometer and an illuminometer; the recorded data are listed in Table 1.

### 2.3. Flexural Strength and Flexural Modulus

The flexural strength (FS) and flexural modulus (FM) were measured after 24 h of storage at 37 °C in distilled water before testing according to the ISO 10477 standard, and a three-point bending test was then carried out using a universal testing machine (EZ-LX, Shimadzu, Kyoto, Japan) (*n* = 20, each group). Before testing, the dimensions of all specimens were determined using a high-precision digital caliper (Mitutoyo Manufacturing, Tokyo, Japan) with an accuracy of 0.01 mm. The specimens were then placed on a support with a 20 mm span distance and loaded with a crosshead speed of 1.0 mm/min until fractured. The maximum force before fracture was recorded in newtons, and the FS (σ) and FM (E) were calculated as follows:

$$\sigma =\frac{3Fl}{2bh^{2}}$$

$$E=\frac{F_{1}l^{3}}{4bh^{3}d}$$

where *F* is the maximum load applied in newtons, *l* is the distance between the supports in millimeters, and *b*, *h*, and d are the width, height, and deflection of the specimen in millimeters, respectively.

### 2.4. Vickers Microhardness

To measure the VHN, the surface of the specimen was polished using 2000-grit SiC paper after printing, followed by post-curing in each intensity group (*n* = 5). The hardness was measured by applying a load of 0.98 N for 15 s using a VHN tester (MMT-X, Matsuzawa, Akita, Japan). One specimen was divided into four equal parts, and post-curing times of 5, 10, and 20 min were accumulated and measured. Three-quarters of the remaining unmeasured area was covered with silver foil to block light exposure to other sections during the test, and the test was repeated three times for each post-curing time according to the intensity, with the average value calculated.

### 2.5. Fourier-Transform Infrared Spectroscopy

To evaluate the DC of the specimen surface, the specimens were polished before post-curing using 2000-grit SiC paper and then post-curing times of 5, 10, and 20 min were accumulated for each intensity group (*n* = 5). Spectra were recorded using Fourier-transform infrared spectroscopy (FT-IR) (Nicolet iS10, Thermo Fisher Scientific, Waltham, MA, USA) with an ATR accessory that featured a diamond crystal on the top plate. ATR-FT-IR spectra were obtained in the 400~4000 cm^−1^ region. All specimens were tested three times with a 4 cm^−1^ resolution and an average of 32 scans per spectrum. The DC was measured by evaluating the absorbance peak intensity of aliphatic bonds around 1638 cm^−1^ and aromatic bonds around 1608 cm^−1^ after baseline correction using spectra collection software (OMNIC, Thermo Fisher Scientific, Waltham, MA, USA). DC was calculated as follows:

$$DC\%=\left[1-\frac{1638{\mathrm{cm}}^{-1}/1608{\mathrm{cm}}^{-1\;}Peak\;height\;\left(cured\right)}{1638{\mathrm{cm}}^{-1/}1608{\mathrm{cm}}^{-1\;}\;Peak\;height\;\left(monomer\right)}\right]\times 100$$

### 2.6. Analysis of 3D Accuracy

The 3D-printed three-unit bridges were post-cured five each according to intensity and time (*n* = 60), and scan data were then acquired in the STL file format using a table-top scanner (T500, Medit, Seoul, Korea), with accuracy within about 7 μm. The scan data were loaded into 3D morphometric analysis software (Geomagic Control X, 3D Systems, Rock Hill, SC, USA), and the scan data were then initially aligned based on the three-unit bridge CAD design data (reference data), and a best-fit algorithm was used to optimize the distance between the meshes of the two data.

For quantitative accuracy analysis, the deviation between the reference and scan data was calculated as the root-mean-square error (RMSE). For qualitative analysis, a color map was used to display deviations within the range of −500 μm (blue color) to 500 μm (red color) and from −10 μm to 10 μm in green color.

### 2.7. Statistical Analysis

The normality of the data in this study was confirmed using the Shapiro–Wilk test. The FS and FM were compared between groups using the Kruskal–Wallis test, followed by the Mann–Whitney test. VHN and DC were checked for significant differences between groups using the Friedman test followed by the Wilcoxon signed-rank test. The significance threshold was set at α = 0.05 for all tests. The statistical analysis was performed using SPSS software (IBM SPSS Statistics ver. 25.0, Chicago, IL, USA).

## 3. Results

### 3.1. Flexural Strength and Modulus

**Figure 2:**
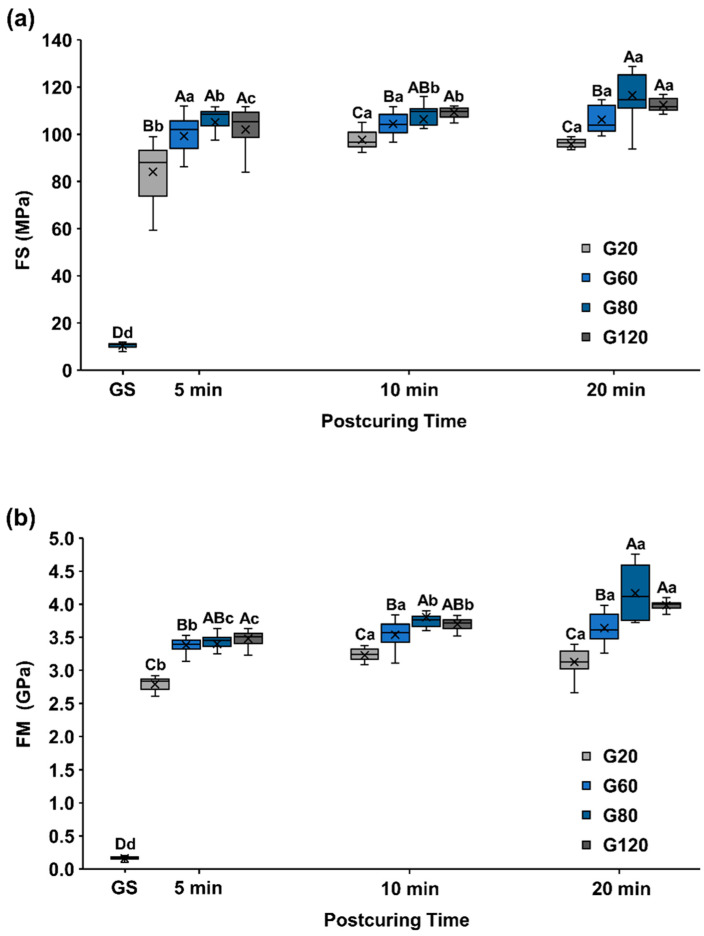
(**a**) Flexural strength (FS). (**b**) Flexural modulus (FM). Uppercase letters indicate significant differences by intensity within the same post-curing time condition using Mann–Whitney with Bonferroni’s correction (*p* < 0.005, A > B > C > D), and lowercase letters indicate statistical differences by post-curing time within the same intensity conditions using Friedman test with Bonferroni’s correction (*p* < 0.0083, a > b > c > d). X mark means the average value of the data.

The FS and FM were measured according to post-curing intensity and time using the three-point bending test method; the data are presented using box plots in with median and mean values in Figure 2 and Table S1.

FS and FM were significant differences according to the intensity and same post-curing time, including GS (*p* < 0.001). The green state (GS: 10.49 ± 0.97 MPa) differed significantly low with the post-curing intensity and time (*p* < 0.001). The specimens post-cured for 5 min had the highest FS in groups G60 (99.24 ± 8.97 MPa), G80 (105.05 ± 7.65 MPa), and G120 (101.98 ± 9.51 MPa) (*p* > 0.005). Conversely, G20 (84.11 ± 11.87 MPa) was significantly low (*p* < 0.001). In 10 min post-cured groups, G80 (106.37 ± 8.50 MPa) and G120 (109.35 ± 2.02 MPa) had the highest FS (p = 0.620), and G20 (97.64 ± 3.98 MPa) was the lowest(p < 0.001). In specimens post-cured for 20 min, FS was highest in group G80 (116.60 ± 8.82 MPa) and G120 (112.39 ± 2.58 MPa) (*p* = 0.049) and followed by G60 (106.24 ± 5.45 MPa) and G20 (95.75 ± 2.86) (*p* < 0.005).

In the FS, within the same intensity group, the specimens post-cured for 5 min had the lowest FS (*p* < 0.001), and there was no significant difference between those post-cured for 10 and 20 min (*p* = 0.547) in group G20. In group G60, no significant differences were found among post-curing time (*p* > 0.0083). The FS in group G80 was also higher for specimens post-cured for 20 min than for those with 5 and 10 min of post-curing (*p* < 0.001). In addition, the FS in group G120 differed significantly followed by 5, 10, and 20 min post-curing (*p* < 0.0083).

In the FM, the specimens cured for 5 min also showed the largest significant differences between groups G80 (3.40 ± 0.22 GPa) and G120 (3.48 ± 0.10 GPa) (*p* = 0.157), while group G60 (3.38 ± 0.10 GPa) followed by group G20 (2.79 ± 0.09 GPa) had significantly lower FMs (*p* < 0.005). There was no significant difference between groups G60 and G80 (*p* = 0.149). Specimens post-cured for 10 min had the highest FM in groups G80 (3.80 ± 0.20 GPa), G120 (3.70 ± 0.09 GPa) (*p* = 0.149) and the group G20 (3.23 ± 0.11 GPa) had the lowest (*p* < 0.001). The specimens cured for 20 min had the highest FM in groups G80 (4.17 ± 0.41 GPa) and G120 (3.99 ± 0.07 GPa), and group G60 (3.64 ± 0.22 GPa) followed by G20 (3.13 ± 0.21 GPa) had significantly smaller differences (*p* < 0.001).

When comparing the difference according to the post-curing time within the same intensity group, the specimens cured for 5 min in groups G20 and G60 had significantly lower FM than the specimens cured for 10 and 20 min (*p* < 0.0083). There was also a significant difference between groups G80 and G120 among 5, 10, and 20 min of post-curing, and the FM increased with the post-curing time (*p* < 0.0083).

### 3.2. Vickers Microhardness

**Figure 3:**
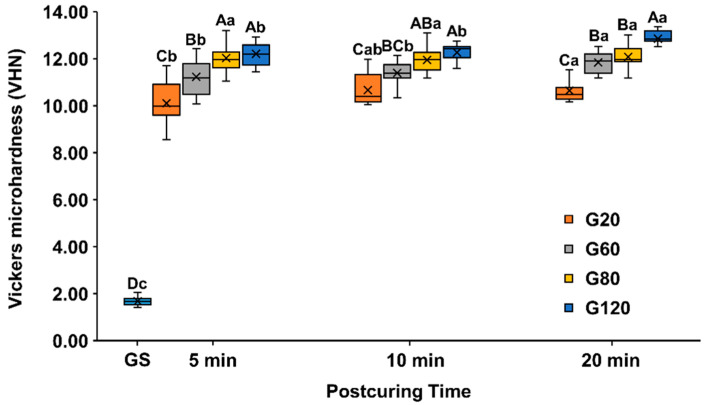
Vickers microhardness. Uppercase letters indicate significant differences by intensity within the same post-curing time condition using Mann–Whitney with Bonferroni’s correction (*p* < 0.005, A > B > C > D), and lowercase letters indicate statistical differences by post-curing time within the same intensity conditions using Wilcoxon test with Bonferroni’s correction (*p* < 0.0083, a > b > c > d). X mark means the average value of the data.

The surface hardness of each specimen was continuously measured by accumulating the post-curing time set in this study, and the data are box plots with median and mean values in Figure 3 and Table S2.

There was a significant difference in VHN according to intensity, including in group GS (1.67 ± 0.18), even within the same post-curing time (*p* < 0.001). The surface hardness of the specimen post-cured for 5 min was the highest in groups G80 (12.03 ± 0.65) and G120 (12.21 ± 0.49) (*p* = 0.305). However, the hardness decreased significantly in group G60 (11.23 ± 0.70) followed by group G20 (10.10 ± 0.65) (*p* < 0.005). In 10 min post-cured groups, G80 (11.94 ± 0.51) and G120 (12.26 ± 0.37) were significantly high (*p* = 0.021), and then they followed by G60 (11.39 ± 0.68) and G20 (10.66 ± 0.61) (*p* < 0.005). After 20 min of post-curing, VHN was highest in group G120 (12.86 ± 0.39), with no difference found between groups G60 (11.84 ± 0.45) and G80 (12.08 ± 0.50) (*p* = 0.345), and lowest for group G20 (10.65 ± 0.46) (*p* < 0.001).

There were differences according to the post-curing time among all intensity groups, including GS (*p* < 0.001). In group G20, VHN was significantly higher for 20 min of post-curing than for 5 min (*p* < 0.0083) and that for 10 min did not differ significantly from that for 5 (*p* = 0.083) or 20 min (*p* = 0.865). Moreover, in groups G60 and G120, VHN was higher for 20 min of post-curing than for 5 or 10 min (*p* < 0.0083), and there was no difference in hardness between 5 and 10 min (G60: *p* = 0.460, G120: *p* = 0.649). On the other hand, there were no differences in group G80 according to post-curing time (*p* > 0.0083).

### 3.3. Fourier-Transform Infrared Spectroscopy

The FT-IR test indicated the DC of the specimen surface, and the data measured continuously according to post-curing times are listed in Table 2.

There was a statistically significant difference in the surface DC according to all the intensity and post-curing times, including in group GS (*p* < 0.05). Furthermore, GS significantly differed in the surface DC according to intensity within the same post-curing time, except at 5 min in group G80 (*p* < 0.0083). The DC of the surface with 5 min of post-curing was the highest in group G60 (*p* < 0.005), and there was no significant difference found in the groups G20 (*p* = 0.744) and G120 (*p* = 0.009) to the intensity within the same post-curing time. In addition, there was no significant difference between the G20 and G120 groups (*p* = 0.106) or between groups G80 and G120 (*p* = 0.098). However, group G20 differed significantly from group G80 (*p* < 0.005). The DC of the surface for 10 min of post-curing was highest in group G20 (*p* < 0.05) compared to G80 (*p* < 0.001). However, there were no differences found between G60 (*p* = 0.187) and G120 (*p* = 0.010). Moreover, there were no significant differences between the intensity groups for 20 min of post-curing (*p* > 0.005).

In addition, the surface DC in groups G60 and G80 did not differ with the post-curing time (*p* > 0.083), while it was highest in groups G20 and G120 for 10 min of post-curing (*p* < 0.083). However, there were no significant differences between 5 and 20 min of post-curing (G20: *p* = 0.140, G120: *p* = 0.609).

### 3.4. Accuracy of the Three-Unit Bridge

**Figure 4:**
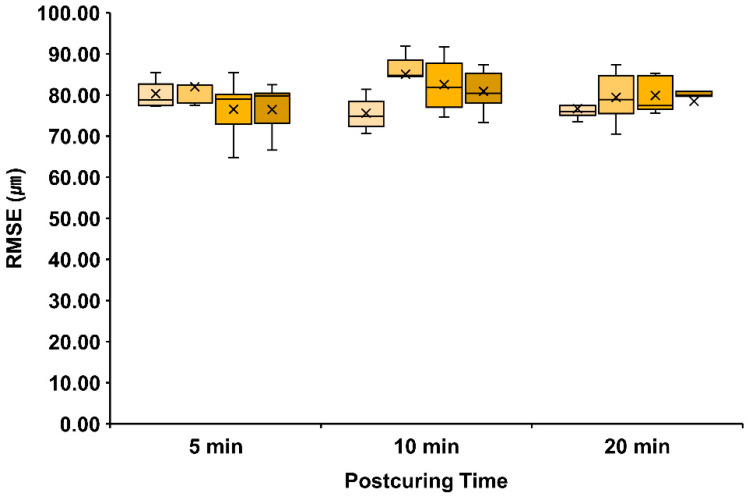
Accuracy of the three-unit bridge. Data are median root-mean-square error (RMSE) values, which did not differ significantly according to the intensity (*p* > 0.005, Mann–Whitney with Bonferroni’s correction) and time of post-curing (*p* > 0.0083, Wilcoxon test with Bonferroni’s correction). X mark means the average value of the data.

The three-unit bridge was used to calculate the mesh deviation using the RMSE according to the intensity and time conditions of post-curing. The median RMSE data are presented in Figure 4 and Table S3 with smaller RMSE values indicating higher accuracy.

RMSE did not differ significantly with the post-curing time within the same intensity groups or according to the intensity group within the same post-curing time (*p* > 0.05). There was no statistically significant difference according to the post-curing time even within the same intensity group (*p* > 0.005). In addition, there was no significant difference according to the intensity group within the same post-curing time (*p* > 0.0083).

**Figure 5:**
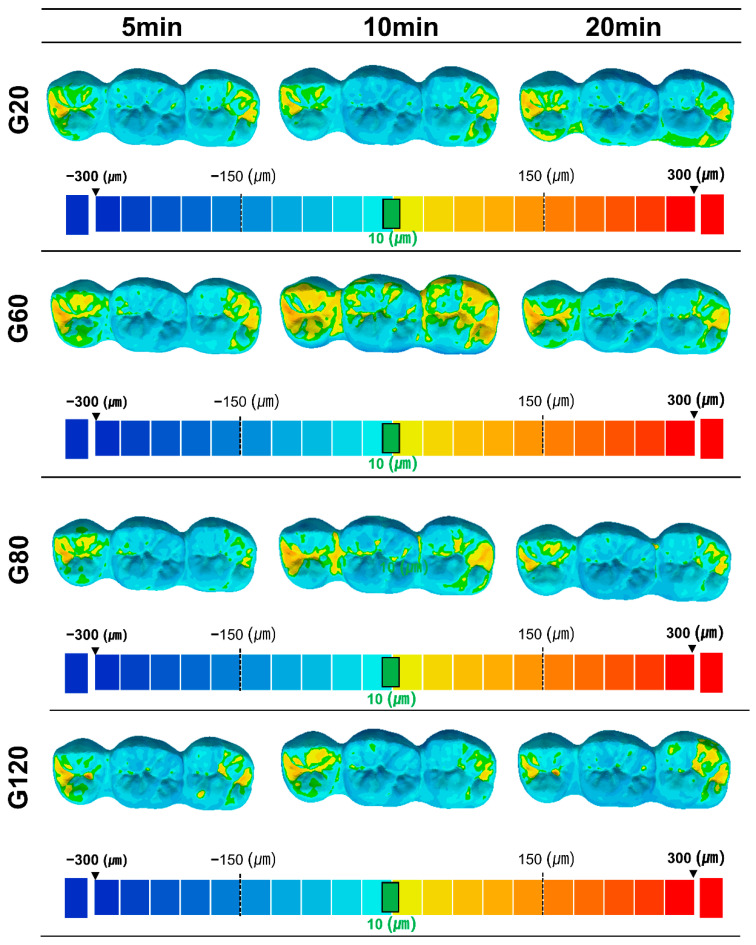
Three-dimensional deviation color map. The display range of positive (red color) and negative (blue color) deviation from 300 to −300 µm, displayed in color to visualize the accuracy. In particular, the area with a deviation within 10 µm is displayed in green.

Qualitatively, analyzing the difference in accuracy using the color map of the analysis program revealed that the volume of the 3D-printed three-unit bridge tended to decrease relative to that in the overall design file. Most of the accuracy difference was at the occlusal surface of the prosthesis, and the accuracy was also comparatively low at the pontic part of the prosthesis (Figure 5).

## 4. Discussion

This study evaluated the effects of light intensity of post-curing equipment and post-curing time on the mechanical properties and dimensional accuracy of interim dental materials. The experiments revealed that FS and VHN differed significantly with the light intensity of the post-curing equipment, while there were no significant differences in the DC and dimensional accuracy of the three-unit bridge. Therefore, the null hypothesis that the light intensity of the post-curing equipment and post-curing time do not affect the flexural properties, VHN, DC, or dimensional accuracy of the three-unit bridge was partially accepted.

In this study, the FS exceeded the ISO 10477 requirement of 50 MPa for crown and veering materials in all groups except for group GS (no post-curing). None of the specimens in group GS broke, and their FS values sharply increased when post-curing was performed. After the same duration of curing, the FS also increased significantly with the light intensity. The FS also tended to increase gradually for longer post-curing times in all groups (G20, G60, G80, and G120), consistent with previous studies that found that the mechanical properties of 3D-printed objects improve, and their anisotropy decreases as the post-curing time increases [9,13,14]. The FM also had a similar tendency to the FS for changes in both light intensity and post-curing time. Characteristically, in cases where the light intensity was higher, even if the post-curing time was short (5 min), the flexural properties were significantly better than in cases where post-curing was performed for longer (20 min) for lower light intensity. This suggests that light intensity had a greater effect on flexural properties than the post-curing time in these experiments.

Interim dental material should function stably in the oral cavity for at least a few days or more than several months during orthodontic treatment or full mouth rehabilitation and should have sufficient wear resistance to maintain the intended occlusion for a long time. A VHN test may be useful for evaluating the wear resistance of the polymer. In this study, the VHN was measured while the post-curing time was accumulated by dividing one specimen into four sections to reduce the deviation for each specimen. Furthermore, to reduce the accumulated light exposure during the measurement process, the non-measured section was covered before the test was conducted. The VHN was found to increase significantly with the light intensity in these experiments. Regarding post-curing time, the VHN was significantly higher after 20 than 5 min of post-curing in all groups except for G60. As with flexural properties, the VHN was higher for the group with high light intensity and 5 min of post-curing than for the group with low light intensity and 20 min of post-curing. This was consistent with the results found by Kim et al. [15] in evaluations of the physical properties, cytotoxicity, and dimensional accuracy for various types of post-curing equipment, who found a relatively high level of surface hardness for a very short post-curing time of 3 s.

The post-curing equipment used in this experiment measured and observed internal temperature changes according to light intensity for 20 min (Table 1). After the start of curing, the temperature increased gradually over 60 s and then maintained relatively constant, with higher light intensities inducing higher average temperatures. Previous studies have found that the diffusion and cross-linking of unreacted monomers increase at high temperatures, leading to more-homogeneous curing and improving mechanical properties [9,16]. The results of the present experiments not only revealed the effect of light intensity itself but also inferred that temperature exerts complex effects. 

DC is the degree to which the monomer converts into a polymer, and insufficient polymerization leaves unreacted monomers, which may affect the mechanical properties and cytotoxicity of the printed product [6,17]. Previous studies have indicated that DC is affected by factors such as exposure time, light wavelength, photo-initiator, and composition of materials such as fillers [18]. Although the minimum clinically acceptable DC has not yet been established, the DC values of conventional light-curing composite resins reportedly vary from 53% to 87% [19,20]. In the present experiments, DC was significantly lower in group GS, and when post-curing was performed, the DC value increased by at least 55% regardless of the light intensity and post-curing time. There was no significant difference between light intensity and post-curing time by the group. This consistency was thought to be attributed to an oxygen inhibition layer existing on the surface since DC was measured only on the superficial layer of each specimen [21]. Tahayeri et al. [22] compared the physical properties of 3D-printed and self-polymerized crown and bridge dental materials. When measuring DC, the specimen was cross-sectioned and ground to a size of 200–300 µm, and a two-dimensional map was obtained with spectra obtained at intervals of 50 µm. No significant difference between 3D-printed resin and conventional materials was found in the printing layer (100 µm). In the 3D-printed material, polymerization was also slightly more advanced in the upper region (near the printing platform) relative to the base region, and DC showed a heterogeneous pattern throughout. The understanding of this could have been improved if inner cross-sections were measured, that is, not limited to the surface.

Low prosthetic accuracy can negatively affect the long-term prognosis of the prosthesis and increase the chair time. Many studies have found that resin shrinkage and deformation occur during and even after post-curing [23,24]. In conventional light-curing composite resins, the deformation caused by resin shrinkage increases with the light intensity, resulting in decreased marginal adaptation. For this reason, previous studies have recommended conducting polymerization with a longer curing time at a lower light intensity [12]. However, using a DLP 3D printing method to perform layer-by-layer polymerization, we thought the results would be different from those for composite resins regarding shrinkage during post-curing. The color maps of the accuracy of the 3D-printed three-unit bridge produced from these experiments revealed that the shrinkage and volumetric deformation of the resin were within 100 µm after the post-curing process, with no significant differences among the light intensity groups. There is no consensus on the clinically acceptable limits of marginal fit in 3D-printed restorations. However, in most previous studies, the marginal discrepancy in conventional FPD has been considered clinically acceptable at ≤120 µm [25,26]. Since the values obtained in these experiments were based on linear measurements of distance, it was difficult to conclude whether the measurements made in this study were clinically acceptable. In addition, Moon et al. [27] found that the accuracy of temporary dental restorations printed with a DLP-type 3D printer deviated within 0.06–0.14 mm for two- and three-unit FPDs, whereas a deviation within 0.13–0.20 mm was observed for six-unit and full-arch types. Therefore, as the number of units of prosthesis increases, the amount of deformation increases, so it seems that more research on objective and clinically acceptable numerical criteria for each is needed. This study found that controlling the light intensity and time of post-curing can be an efficient method to obtain appropriate mechanical properties and accuracy. As 3D printing technology increases in clinical dental practice and various types of equipment and materials are used, it is necessary to consider and understand the optimal post-curing conditions that can affect the outcome. This study had several limitations. First, it was difficult to accurately analyze the composition of the used interim dental material. Although only one material was used in this experiment, the additives of photo-initiator, filler, and matrix differ between different types of resin and different manufacturers. These differences in composition also affected the results, and it is expected that a broader understanding will be achieved if a follow-up study is conducted. Second, the post-curing equipment used in this study was divided into upper and lower plates that were cured at the same time. However, various parameters of the UV light, such as the irradiation direction, distance, and wavelength, may vary between different post-curing devices, so further follow-up studies will be helpful.

## 5. Conclusions

Within the limitations of this study, the following conclusions can be drawn: (1) The flexural properties and VHN increased significantly with the light intensity of the post-curing equipment. (2) The flexural properties and VHN increased with the post-curing time. (3) The flexural properties and VHN were similar or higher for curing for a short time at a high light intensity than for a long time at a low light intensity. (4) The 3D accuracy of a three-unit bridge was not significantly affected by the light intensity or post-curing time.

## Figures and Tables

**Table 1:** Intensity and temperature of post-curing equipment.

Group	G20	G60	G80	G120
Intensity (mW/cm^2^)	1.4–1.6	2.2–3.0	3.8–4.4	6.4–7.0
Temperature (°C)	35.1–42.0	36.8–4.43	39.7–52.9	41.4–61.3

**Table 2:** Surface degree of conversion of the specimen for post-curing intensity and time (mean ± SD%).

	GS	5 min	10 min	20 min
GS	52.81 ± 2.19 ^b^	52.81 ± 2.19 ^C^		
G20		59.61 ± 2.67 ^AB,b^	63.04 ± 3.94 ^A,a^	57.18 ± 3.74 ^A,b^
G60		59.99 ± 1.93 ^A,a^	61.07 ± 3.47 ^AB,a^	59.91 ± 4.41 ^A,a^
G80		55.83 ± 3.73 ^B,a^	58.61 ± 1.90 ^B,a^	57.13 ± 2.87 ^A,a^
G120		58.19 ± 1.53 ^AB,b^	60.09 ± 2.54 ^AB,a^	57.76 ± 3.26 ^A,b^

Uppercase letters indicate significant differences according to intensity within the same post-curing time using Mann–Whitney with Bonferroni’s correction (*p* < 0.005, A > B > C), and lowercase letters indicate significant differences according to post-curing time within the same intensity using Wilcoxon test with Bonferroni’s correction (*p* < 0.0083, a > b > c).

## Data Availability

The data presented in this study are available on request from the corresponding author.

## Supplementary Materials

The following supporting information can be downloaded at: www.mdpi.com/article/10.3390/ma15196889/s1, Table S1: Mean ± SD values on flexural strength and modulus; Table S2: Mean ± SD values on Vickers microhardness; Table S3: Mean ± SD RMSE values on accuracy of the three-unit bridge.

## References

[1] Stansbury J.W., Idacavage M.J. (2016). 3d printing with polymers: Challenges among expanding options and opportunities. Dent. Mater..

[2] Baumers M., Dickens P., Tuck C., Hague R. (2016). The cost of additive manufacturing: Machine productivity, economies of scale and technology-push. Technol. Forecast. Soc. Change.

[3] Barazanchi A., Li K.C., Al-Amleh B., Lyons K., Waddell J.N. (2017). Additive technology: Update on current materials and applications in dentistry. J. Prosthodont..

[4] Beuer F., Schweiger J., Edelhoff D. (2008). Digital dentistry: An overview of recent developments for cad/cam generated restorations. Br. Dent. J..

[5] Revilla-León M., Özcan M. (2019). Additive manufacturing technologies used for processing polymers: Current status and potential application in prosthetic dentistry. J. Prosthodont..

[6] Calheiros F.C., Daronch M., Rueggeberg F.A., Braga R.R. (2008). Degree of conversion and mechanical properties of a bisgma: Tegdma composite as a function of the applied radiant exposure. J. Biomed. Mater. Res. Part B-Appl. Biomater..

[7] Alharbi N., Osman R.B., Wismeijer D. (2016). Factors influencing the dimensional accuracy of 3d-printed full-coverage dental restorations using stereolithography technology. Int. J. Prosthodont..

[8] Shim J.S., Kim J.E., Jeong S.H., Choi Y.J., Ryu J.J. (2020). Printing accuracy, mechanical properties, surface characteristics, and microbial adhesion of 3d-printed resins with various printing orientations. J. Prosthet. Dent..

[9] Bayarsaikhan E., Lim J.-H., Shin S.-H., Park K.-H., Park Y.-B., Lee J.-H., Kim J.-E. (2021). Effects of postcuring temperature on the mechanical properties and biocompatibility of three-dimensional printed dental resin material. Polymers.

[10] Reymus M., Lümkemann N., Stawarczyk B. (2019). 3d-printed material for temporary restorations: Impact of print layer thickness and post-curing method on degree of conversion. Int. J. Comput. Dent..

[11] Bayarsaikhan E., Gu H., Hwangbo N.-K., Lim J.-H., Shim J.-S., Lee K.-W., Kim J.-E. (2022). Influence of different postcuring parameters on mechanical properties and biocompatibility of 3d printed crown and bridge resin for temporary restorations. J. Mech. Behav. Biomed. Mater..

[12] Unterbrink G.L., Muessner R. (1995). Influence of light intensity on two restorative systems. J. Dent..

[13] Kim D., Shim J.-S., Lee D., Shin S.-H., Nam N.-E., Park K.-H., Shim J.-S., Kim J.-E. (2020). Effects of post-curing time on the mechanical and color properties of three-dimensional printed crown and bridge materials. Polymers.

[14] Aati S., Akram Z., Shrestha B., Patel J., Shih B., Shearston K., Ngo H., Fawzy A. (2022). Effect of post-curing light exposure time on the physico–mechanical properties and cytotoxicity of 3d-printed denture base material. Dent. Mater..

[15] Kim J.H., Kwon J.S., Park J.M., Lo Russo L., Shim J.S. (2022). Effects of postpolymerization conditions on the physical properties, cytotoxicity, and dimensional accuracy of a 3d-printed dental restorative material. J. Prosthet. Dent..

[16] Jindal P., Juneja M., Bajaj D., Siena F.L., Breedon P. (2020). Effects of post-curing conditions on mechanical properties of 3d printed clear dental aligners. Rapid Prototyp. J..

[17] Fujioka-Kobayashi M., Miron R.J., Lussi A., Gruber R., Ilie N., Price R.B., Schmalz G. (2019). Effect of the degree of conversion of resin-based composites on cytotoxicity, cell attachment, and gene expression. Dent. Mater..

[18] Obici A.C., Sinhoreti M.A.C., Frollini E., Sobrinho L.C., de Goes M.F., Henriques G.E.P. (2006). Monomer conversion at different dental composite depths using six light-curing methods. Polym. Test..

[19] Silikas N., Eliades G., Watts D.C. (2000). Light intensity effects on resin-composite degree of conversion and shrinkage strain. Dent. Mater..

[20] Xu T., Li X., Wang H., Zheng G., Yu G., Wang H., Zhu S. (2020). Polymerization shrinkage kinetics and degree of conversion of resin composites. J. Oral Sci..

[21] Studer K., Decker C., Beck E., Schwalm R. (2003). Overcoming oxygen inhibition in uv-curing of acrylate coatings by carbon dioxide inerting, part i. Prog. Org. Coat..

[22] Tahayeri A., Morgan M., Fugolin A.P., Bompolaki D., Athirasala A., Pfeifer C.S., Ferracane J.L., Bertassoni L.E. (2018). 3d printed versus conventionally cured provisional crown and bridge dental materials. Dent. Mater..

[23] Park J.-M., Jeon J., Koak J.-Y., Kim S.-K., Heo S.-J. (2021). Dimensional accuracy and surface characteristics of 3d-printed dental casts. J. Prosthet. Dent..

[24] Shin S.-H., Doh R.-M., Lim J.-H., Kwon J.-S., Shim J.-S., Kim J.-E. (2021). Evaluation of dimensional changes according to aging period and postcuring time of 3d-printed denture base prostheses: An in vitro study. Materials.

[25] Kaleli N., Sarac D. (2017). Influence of porcelain firing and cementation on the marginal adaptation of metal-ceramic restorations prepared by different methods. J. Prosthet. Dent..

[26] Pompa G., Di Carlo S., De Angelis F., Cristalli M.P., Annibali S. (2015). Comparison of conventional methods and laser-assisted rapid prototyping for manufacturing fixed dental prostheses: An in vitro study. Biomed Res. Int..

[27] Moon W., Kim S., Lim B.-S., Park Y.-S., Kim R.J.-Y., Chung S.H. (2021). Dimensional accuracy evaluation of temporary dental restorations with different 3d printing systems. Materials.

