# Supplementary material for: Effects of Post-Curing Light Intensity on the Mechanical Properties and Three-Dimensional Printing Accuracy of Interim Dental Material
Source: Materials (Basel). 2022 Oct 4;15(19):6889. doi: 10.3390/ma15196889 (PMC9570601; doi:10.3390/ma15196889)
Supplement: Supplementary file 1 [file materials-15-06889-s001.zip › materials-1929662-supplementary.pdf]

materials

MDPI

Supplementary

Effects of Post-curing Light Intensity on the Mechanical Properties and Three-Dimensional Printing Accuracy of Interim Dental Material

Min-Jung Kang †, Jung-Hwa Lim †, Chan-Gyu Lee, and Jong-Eun Kim *

Table S1. Mean ± SD values on flexural strength and modulus.

Group	Post-Curing time	Flexural Strength (MPa)	Flexural Modulus (GPa)
GS	-	10.49 ± 0.97	0.16 ± 0.02
G20	5min	84.11 ± 11.87	2.79 ± 0.09
	10min	97.64 ± 3.98	3.23 ± 0.11
	20min	95.75 ± 2.86	3.13 ± 0.21
G60	5min	99.24 ± 8.97	3.38 ± 0.10
	10min	104.42 ± 4.60	3.54 ± 0.20
	20min	106.24 ± 5.45	3.64 ± 0.22
G80	5min	105.05 ± 7.65	3.40 ± 0.22
	10min	106.37 ± 8.50	3.80 ± 0.20
	20min	116.60 ± 8.82	4.17 ± 0.41
G120	5min	101.98 ± 9.51	3.48 ± 0.10
	10min	109.35 ± 2.02	3.70 ± 0.09
	20min	112.39 ± 2.58	3.99 ± 0.07

Table S2. Mean ± SD values on Vickers microhardness

Group	Post-Curing time	Surface Hardness (VHN)
GS	-	1.67 ± 0.18
G20	5min	10.10 ± 0.65
	10min	10.66 ± 0.61
	20min	10.65 ± 0.46
G60	5min	11.23 ± 0.70
	10min	11.39 ± 0.68
	20min	11.84 ± 0.45
G80	5min	12.03 ± 0.65
	10min	11.94 ± 0.51
	20min	12.08 ± 0.50
G120	5min	12.21 ± 0.49
	10min	12.26 ± 0.37
	20min	12.86 ± 0.39

Table S3. Mean ± SD RMSE values on the accuracy of the three-unit bridge.

Group	Post-Curing time	RMSE (μm)
**G20**	5min	80.34 ± 3.62
	10min	75.56 ± 4.39
	20min	76.66 ± 2.96
**G60**	5min	82.00 ± 4.84
	10min	85.06 ± 6.09
	20min	79.40 ± 6.83
**G80**	5min	76.48 ± 7.92
	10min	82.58 ± 7.15
	20min	79.90 ± 4.66
**G120**	5min	76.50 ± 6.54
	10min	80.88 ± 5.63
	20min	78.44 ± 5.92
